# Supplementary material for: The bHLH Transcription Factor OsbHLH057 Regulates Iron Homeostasis in Rice
Source: Int J Mol Sci. 2022 Nov 28;23(23):14869. doi: 10.3390/ijms232314869 (PMC9739582; doi:10.3390/ijms232314869)
Supplement: Supplementary file 1 [file ijms-23-14869-s001.zip › Supplemental Figures.pdf]

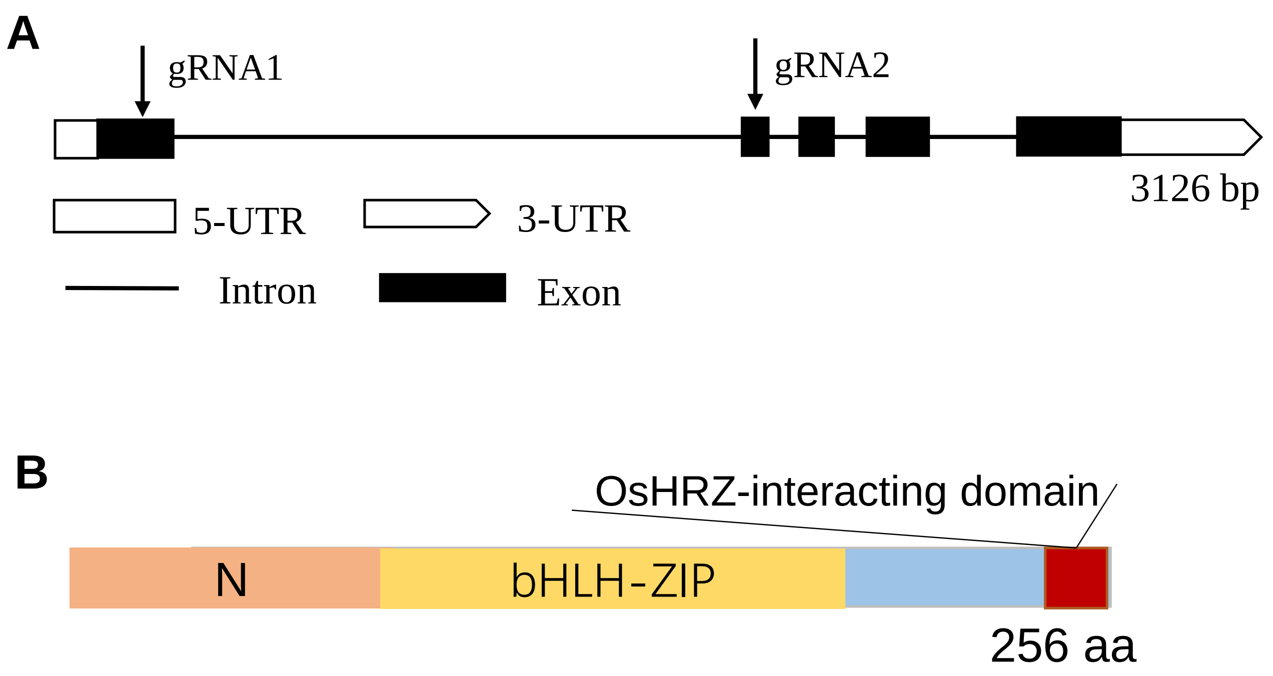

Figure S1. *OsbHLH057* gene structure and its protein encoding structure. (**A**) Diagram of the *OsbHLH057* gene structure. (**B**) Diagram of the OsbHLH057 protein structure. gRNA, guide RNA; aa, amino acids.

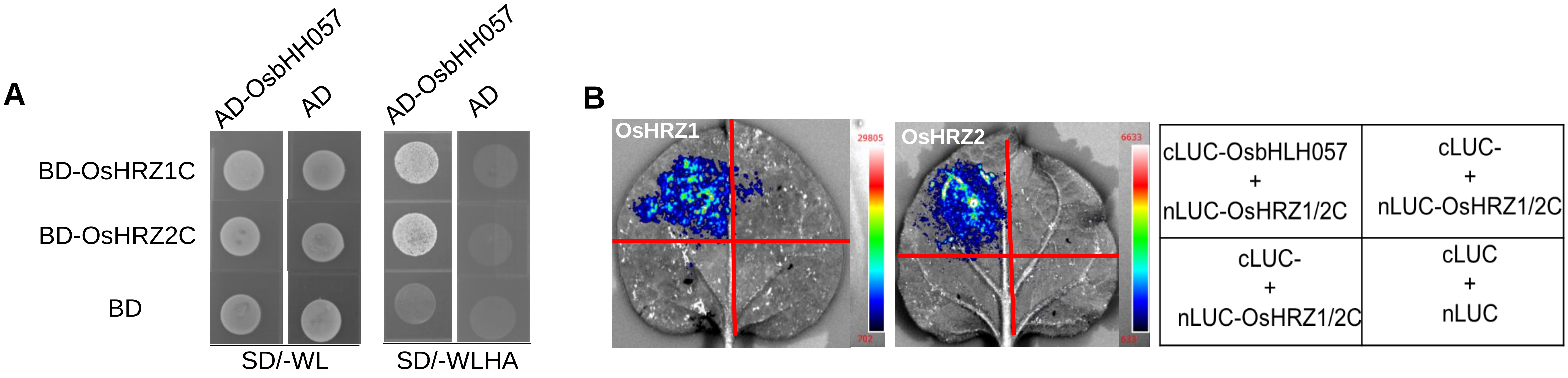

Figure S2. OsbHLH057 is an interact partner of OsHRZ1 and OsHRZ2.

(**A**) OsbHLH057 interacts with OsHRZ1 and OsHRZ1 in yeast cells. The C-terminal part of OsHRZ1 and OsHRZ2 were fused with the GAL4 DNA binding domain (BD) and OsbHLH057 with the GAL4 activation domain (AD) into *pGBKT7* and *pGADT7*, respectively. The different combination vectors were transferred into AH109 yeast strain, and then the different yeast strains were plated on nonselective medium without tryptophan (W) and leucine (L) or on selective medium without W, L, histidine (H), and ade (A). (**B**) OsbHLH057 interacts with OsbHLH057 in plant. The C-terminal part of OsHRZ1 and OsHRZ2 were fused with the N-terminal part of LUC (nLUC) and OsbHLH057 with the C-terminal part of LUC (cLUC) into expression vectors prior to transfer into tobacco leaves. nLUC and cLUC alone were used as negative controls. The different combinations were shown in form which in the right part of picture.

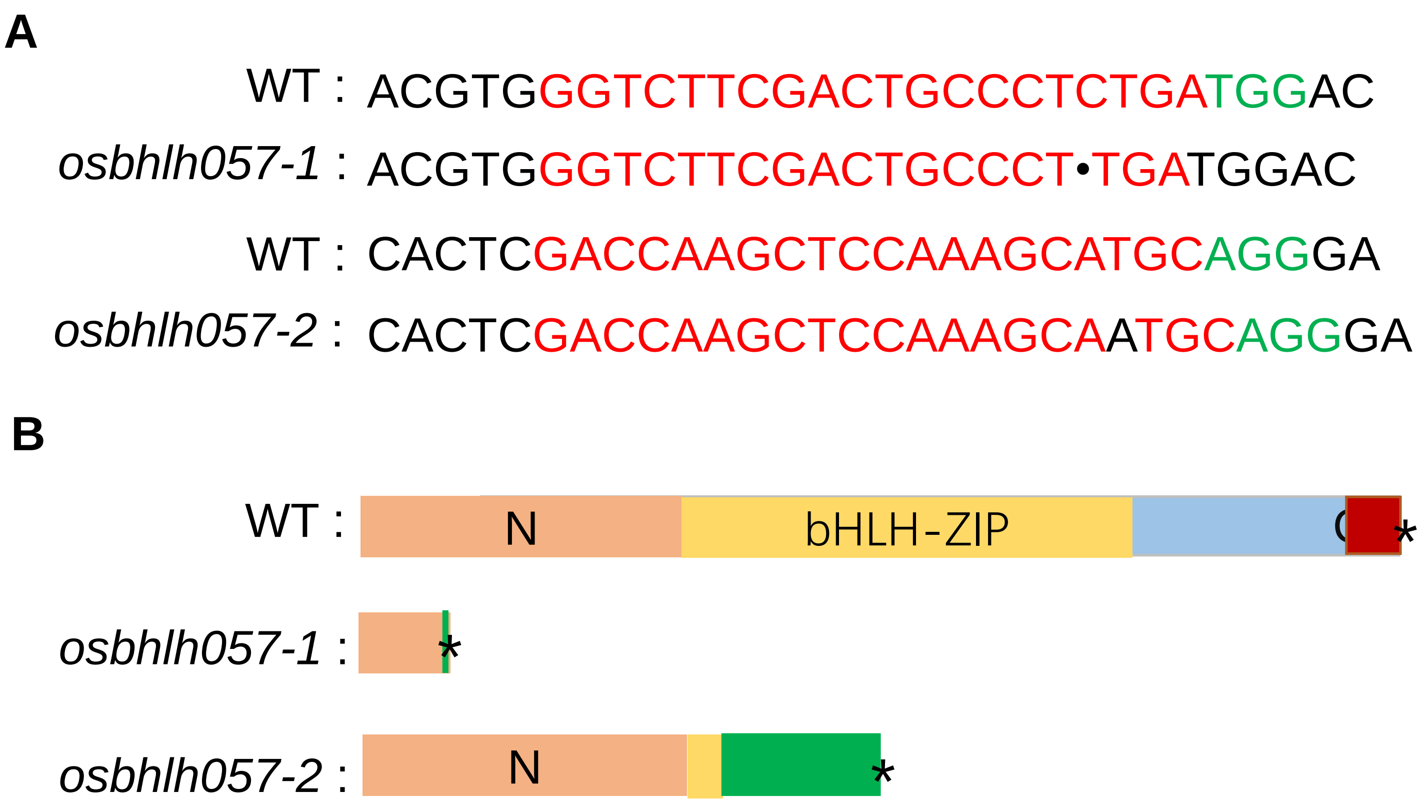

Figure S3. Gene editing of *OsbHLH057*. (**A**) The results of sequence from *osbhlh057-1* and *osbhlh057-2*. *osbhlh057-1* harbor a deletion of 'C' and *osbhlh057-2* contains an insertion of 'A'. (**B**) The diagram of OsbHLH057 and the predicted mutated proteins of *osbhlh057* mutants. Green boxes represent mutation segments with frameshift; * represents premature stop codon. WT, wild type; *osbhlh057-1* and *osbhlh057-2* are two independent lines of *OsbHLH057* knockout mutations.

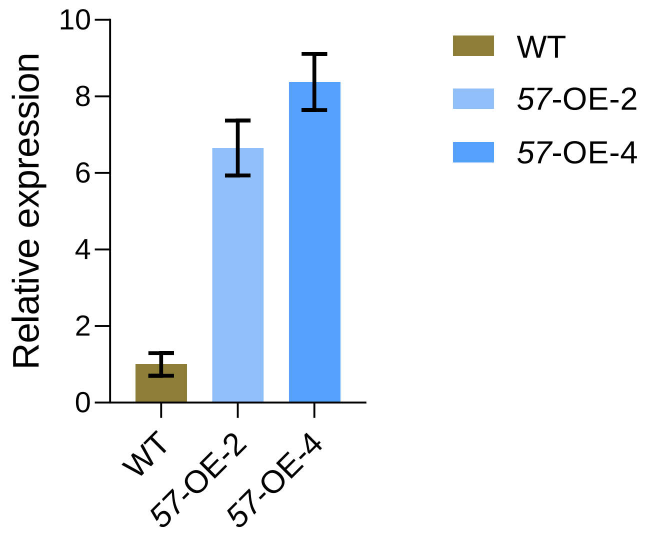

Figure S4. The relative expression of *OsbHLH057* in the WT and *OsbHLH057* overexpression plants. RT-qPCR analysis of the expression levels of *OsbHLH057* in the root of the WT and *OsbHLH057* overexpression plants. *OsActin1* was used as the internal standard. Data are given as the means ±SD of three biological replicates. WT, wild type; *57*-OE-2, *OsbHLH057*-OE-2; *57*-OE-4, *OsbHLH057*-OE-4.
